# Is Chorea a Sequence of Rheumatism?

**Published:** 1875-11

**Authors:** J. C. Bishop

**Affiliations:** Middleport, Ohio


### IS CHOREA A SEQUENCE OF RHEUMATISM?

BY J. C. BISHOP, M. D., MIDDLEPORT, OHIO.

It will be unnecessary to more than mention the fact that certain diseases engender a predisposition to certain other diseases, which may be of far greater gravity, sometimes at least, than the disease which engenders it; nor that well marked and definite conditions supervene upon other conditions in far too constant proportion to be considered in anywise accidental; but that, in fact, certain diseased conditions beget sequellæ, which are, in the present state of medical knowledge, confidently predicted and observed; e.g. in pleuritis effusion may occur, and if the disease lasts a definite length of time, may, with almost absolute certainty, be predicted. Following rheumatism, pericarditis may frequently be observed, as sequellæ, or after results of certain morbid conditions not yet thoroughly understood. In this connection, we desire to raise the question, which has been gradually forced upon us, with certain convictions, warranted by our interpretation of clinical facts occurring under our immediate observation, is chorea a sequence of rheumatism? The pathological conditions existing in both these diseases are somewhat questionable, and in chorea, we may assert that nothing whatever is definitely known regarding its causation. Therefore, as we are to deal with an abstruse problem, whatever truths we may be able to demonstrate or suggest, we must at last fall back upon the axiom, that "there is nothing new under the sun," and that all truth exists potentially in every mind. Both rheumatism and chorea are readily reorganized by the peculiarities belonging to them respectively; the former is a very common disease, while the latter is somewhat rare; the former has certain sequellæ, which, though not constant, have been demonstrated to exist in such a proportion of cases as to preclude the idea of being accidental or intercurrent. The latter has not yet exhibited a tendency to become procurative of sequellæ, and no sequences are observed peculiar to itself as a primary disease, while the lesions, discovered after death from chorea, are exactly such as we know to be observed after death from rheumatism.

It being true, also, that in at least four-fifths of the cases of chorea that occur, a previous history of rheumatism exists, we must in the absence of any other, and positive clinical facts bearing upon its causation, conclude that it is dependent upon rheumatism; nor is the fact that all patients who have rheumatism do not have chorea, a valid argument against the conclusion, more than that all persons who have had rheumatism do not have heart disease, argue that the former does not tend to beget the latter. Yet, the same may be true of this as of other diseases, namely, that for its development, the conditions favorable must be present, but what the conditions are is not so readily determined. Chorea is said, by Professor Flint, to be probably caused by the presence of worms in the intestinal canal, fright, anger, etc., while there is abundant reason to believe that the development of the affection is formed by anæmia. That chorea may very rarely be caused by the means of worms, fright and anger, etc., seems to be true, but it will evidently be the fewest number of cases indeed, while anæmia may certainly cause it by being that peculiar anæmia resulting from a protracted rheumatism, we are ready to admit, but pure anæmia, dependent upon no definite pathological conditions as a cause of chorea, we strongly doubt. M. See has been led, by extended researches, to the conclusion that the affection is to be considered as essentially rheumatic. If such a connection be true, it would go far to support the neurotic origin of the latter disease; and, indeed, there is more than a possibility that this is true.

Dr. Henri Roger, Physician to the Children's Hospital, of Paris, deduces, from a collection of cases, the conclusion, that there exists a pathological connection between chorea, rheumatism and eudecarditis. Dr. John W. Ogle, of London, (*British and Foreign Medical Chirurgical Review, January*, 1868,) found in ten out of sixteen fatal cases of chorea, fibrinous deposit or granulations existing upon the valves, or some portion of the lining membrane of the heart. Here are found the identical conditions after death from chorea, which are found in death from rheumatism, and if such appearances belong to both these diseases respectively, and chorea has no peculiar features to claim as its own, we must naturally conclude as to the resemblance of the diseases under consideration—their identity or their dependence of one upon the other—and since rheumatism precedes the chorea, must engender the conditions which are favorable to its advent. That chorea is rheumatic, we are not prepared to believe, only so far as that the post-mortem appearances are identical for the most part, and that the former is the legitimate result of the latter, under favorable circumstances, for its development, in so much that chorea lacks the essential elements of rheumatism, since there is no pain, or but little, no redness, no swelling, and no increased heat—in fact, it is devoid of any of the essentials of inflammation, and the products of inflammation, rheumatic in character, which are found in post-mortems of chorea, date from the rheumatic attack, and play an important part in the production of choreic phenomena. The presence of eudocardial murmurs is frequently observed in chorea, and takes place only in those cases which have been preceded by rheumatism; for we have no data, by which to endow chorea with the power of inducing such conditions, of the number of cases of chorea witnessed, five or six occurring in the practice of your reporter; a previous history of rheumatism was fairly and unequivocally established in all but one, and in that there was a doubt. One case, occurring only a few months since, began to evince choreic symptoms within a month after the subsidence of the rheumatism, and as a matter worthy of note, the choreic movements are limited almost exclusively to the left side of the arm, trunk and leg in which the phenomena of rheumatism were most severe and obstinate. We suffered, at the age of 13, from a severe and prolonged attack of acute rheumatism, and quickly supervening an attack of chorea, which lasted for nearly two years before it entirely disappeared. We have seen no case of chorea in hale, hearty, muscular subjects, and believe that under such circumstances it is rarely seen; while on the other hand, it attacks weakly and debilitated persons who have probably undergone an attack of rheumatism and received the conditions favorable. Whether this may be from a change in the blood itself from the contamination of that fluid by the products of rheumatism, or by the impairment of the nervous system, begetting a spasmodic and irregular muscular action, is not clear. The position taken in this paper, is that guaranteed by clinical observation, and warranted by facts, as they appear in the practice of not myself alone, but of others.

